# 3D-Printed Nanocellulose-Based Cushioning–Antibacterial Dual-Function Food Packaging Aerogel

**DOI:** 10.3390/molecules26123543

**Published:** 2021-06-10

**Authors:** Wei Zhou, Jiawei Fang, Shuwei Tang, Zhengguo Wu, Xiaoying Wang

**Affiliations:** State Key Laboratory of Pulp and Paper Engineering, South China University of Technology, 381 Wushan Road, Tianhe District, Guangzhou 510640, China; annzhouw@outlook.com (W.Z.); fangjw010@163.com (J.F.); T13061427625@163.com (S.T.); xyw@scut.edu.cn (X.W.)

**Keywords:** 3D printing, carboxymethyl nanocellulose, silver nanoparticles, cushioning, antibacterial

## Abstract

Cushioning and antibacterial packaging are the requirements of the storage and transportation of fruits and vegetables, which are essential for reducing the irreversible quality loss during the process. Herein, the composite of carboxymethyl nanocellulose, glycerin, and acrylamide derivatives acted as the shell and chitosan/AgNPs were immobilized in the core by using coaxial 3D-printing technology. Thus, the 3D-printed cushioning–antibacterial dual-function packaging aerogel with a shell–core structure (CNGA/C–AgNPs) was obtained. The CNGA/C–AgNPs packaging aerogel had good cushioning and resilience performance, and the average compression resilience rate was more than 90%. Although AgNPs was slowly released, CNGA/C–AgNPs packaging aerogel had an obvious antibacterial effect on *E. coli* and *S. aureus*. Moreover, the CNGA/C–AgNPs packaging aerogel was biodegradable. Due to the customization capabilities of 3D-printing technology, the prepared packaging aerogel can be adapted to more application scenarios by accurately designing and regulating the microstructure of aerogels, which provides a new idea for the development of food intelligent packaging.

## 1. Introduction

During the transportation, fruits and vegetables are easily damaged by mechanical injury due to static pressure, shock, vibration and collision, which lead to irreversible quality loss [1,2]. According to statistics, the loss rate of fruits caused by mechanical damage in the transportation process is as high as 30% [3]. However, the commonly used foamed polystyrene (EPS) and polyethylene (EPE) materials are hard to be degraded and recycled [4]. Therefore, it is urgent to develop an environment-friendly biodegradable cushioning packaging material for fruits and vegetables. Nanocellulose possesses the advantages of being biodegradable, renewable, safe, and non-toxic, and the high aspect ratio gives it better strength and toughness, which can be used as the matrix of cushioning packaging materials [5,6,7].

In the process of storage and transportation of fruits and vegetables, in addition to shock absorption and cushioning, there is another huge demand for antibacterial packaging [8]. Traditional cushioning packaging has almost no antibacterial activity, while the cushioning packaging with antibacterial function can effectively control the quality degradation of fruits and vegetables caused by the growth and reproduction of epidermal microorganisms after harvest, synergistically reducing the loss rate of fruits and vegetables [9]. Silver nanoparticles (AgNPs), a kind of broad-spectrum antibacterial agent, have been widely used in many fields, including food packaging [10,11,12], which could be used as a functional component to endow the cushioning food packaging materials with antibacterial activity. However, it is worth noting that there is a cumulative toxicity risk of silver nanoparticles, and its release needs to be controlled for reducing the potential harm to the human body and ecology during the applications [13,14].

Coaxial 3D-printing technology has the ability to precisely design and construct fine structures of materials, which can be used to fabricate the shell–core structure for the immobilization of AgNPs in one step. More importantly, the customization capabilities of coaxial 3D-printing technology can accurately design and regulate the microstructure of materials according to different application scenarios [15], so as to obtain the most suitable customized structure and the best shock absorption and cushioning performance of the packaging material. Meanwhile, the highly programmed characteristics endow 3D-printed antibacterial and cushioning dual-function food packaging materials with the potential of large-scale industrialization.

**Figure 1:**
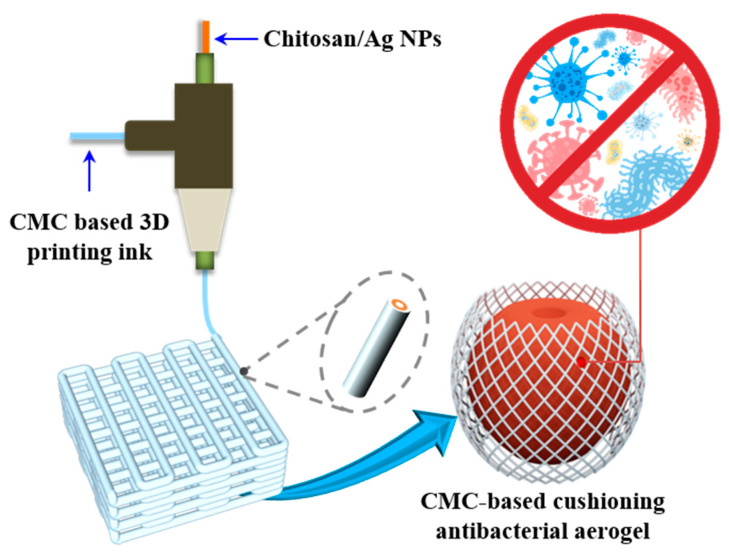
Scheme for the preparation process of 3D-printed carboxymethyl nanocellulose-based cushioning and antibacterial dual-function food packaging aerogels.

Therefore, as shown in Figure 1, carboxymethyl nanocellulose (CMC) is used as the matrix, which is supplemented with acrylamide derivatives and glycerol to obtain CMC-based 3D-printing ink. With that ink as the shell material, chitosan/sliver nanoparticles (Cts/AgNPs) as the core layer, the biodegradable carboxymethyl nanocellulose-based antibacterial and cushioning dual functional food packaging aerogels were prepared by coaxial 3D-printing technology.

## 2. Results and Discussion

### 2.1. Rheological Performances of 3D-Printing Inks

**Figure 2:**
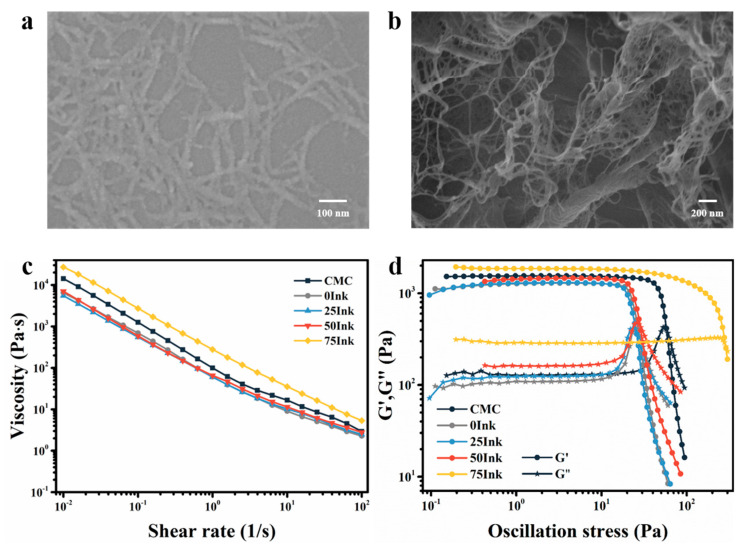
SEM images of CMC (**a**) before and (**b**) after freeze drying. (**c**) Viscosity as a function of the shear rate for CMC and CMC-based inks, (**d**) G′ and G″ as a function of the shear stress for CMC and CMC-based inks.

The microscopic morphology of carboxymethyl nanocellulose (CMC) was characterized by SEM, and the results are shown in Figure 2a,b. The diameter of CMC was in the nanoscale range with a large aspect ratio. After freeze drying, the fibers were entangled to form a three-dimensional network structure, indicating that CMC is suitable for the construction of cushioning packaging aerogels.

Rheological performance is a key factor affecting the printability and fidelity of 3D-printing inks. In this study, the rheological performances of CMC and CMC-based 3D-printing inks were analyzed. The results are shown in Figure 2c,d. As can be seen from Figure 2c, CMC-based 3D-printing inks retained the shear thinning behavior of CMC. When the shear rate increased from 10^−2^ s^−1^ to 10^2^ s^−1^, the viscosity of CMC and CMC-based 3D-printing inks generally decreased by three orders of magnitude. The shear thinning behavior of CMC-based 3D-printing inks could reduce the extrusion pressure needed in the printing process, so that the inks could be extruded more smoothly from the needle, especially the coaxial needle. As can be seen from Figure 2c, with the increase of glycerol concentration in the ink, its viscosity gradually increased. The viscosity of 75Ink was up to 26,854.14 Pa at 10^−2^ s^−1^. When the shear force was removed, CMC-based 3D-printing inks had a higher viscosity at low shear rates, which helped to maintain the shape after printing. As can be seen from Figure 2d, with the increase of glycerin content, the storage modulus, loss modulus, and yield stress of CMC-based 3D-printing inks were all improved, which promoted the ink to have higher printing fidelity in the 3D-printing process. In addition, the yield stress of 75Ink was the largest, reaching 275.30 Pa.

### 2.2. Effect of Glycerol Content on 3D-Printed Cushioning Aerogels

**Figure 3:**
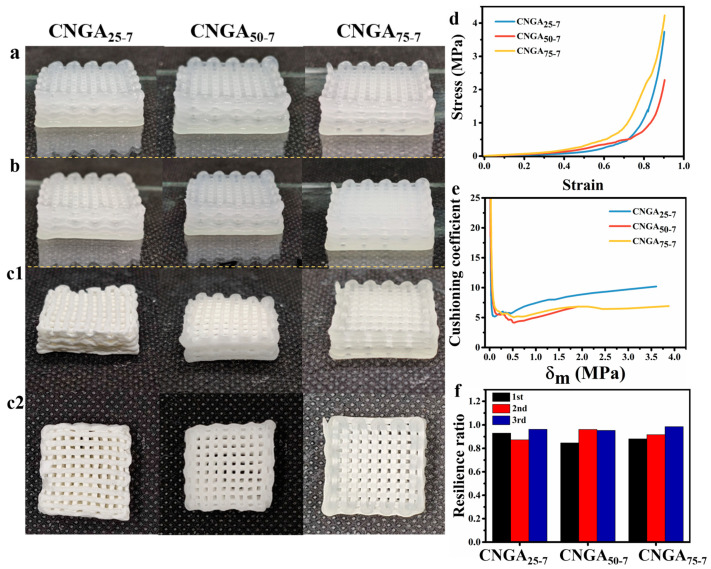
Morphology of 3D-printed samples by CMC-based inks with different glycerin content (**a**) before and (**b**) after UV curing and (**c1**,**c2**) after freeze drying. (**d**) Stress–strain curve, (**e**) cushioning curves, and (**f**) thrice compression resilience ratio of samples with different glycerin content.

The morphology and microstructure of CMC-based 3D-printed cushioning aerogels prepared by uniaxial 3D printing were preliminarily analyzed. As shown in Figure 3, CMC-based inks with 25 vol% (25Ink), 50 vol% (50Ink), and 75 vol% (75Ink) glycerin solutions all had excellent printing performance, and the printed samples presented regular network structures with distinct layers. There was no deformation or collapse in the high-fidelity printing process. After UV curing, the surfaces of the aerogels appeared misty due to cross-linking. Different from the wet morphology of the samples, the texture and structures of the printed samples with different glycerol content showed a great difference after freeze drying.

As can be clearly seen from Figure 3a–c, with the increase of glycerin content, the texture of aerogel gradually changed from white, loose, and porous spongy to translucent gelatinous, and the shape retention ability of dry aerogels were enhanced. After freeze-drying, the sample with 75% glycerin (CNGA_75-7_) could completely keep the regular grid structure formed by 3D printing, while other samples showed different degrees of shrinkage and deformation.

In order to further study the difference of the printed samples with different glycerol content, their microstructures were characterized by SEM. As can be seen from Figure S1a2, the printed sample by 25Ink (CNGA_25-7_) presented a loose and porous structure with large channels, and the lumps produced by the material fragmentation during the freeze-drying process were attached to its surface. Compared with the CNGA_25-7_, the CNGA_50-7_ had a dense structure, but obvious surface wrinkle lines can be observed (Figure S1b2), while the CNGA_75-7_ had a smooth and denser surface after freeze drying. Therefore, as shown in Figure S1, with the increase of glycerol concentration in the printing ink, the surface micrograph of the obtained samples showed an obvious densification trend, which was conducive to maintaining the shape of the printed samples after freeze drying.

In addition, the differences of static compression performance and elastic recovery energy of samples with different glycerol contents have also been investigated (Figure 3). As can be seen from Figure 3d, with the increase of glycerin content, the elastic modulus and strength of the aerogel gradually increased, indicating that glycerin helped to improve the compression resistance of the printed aerogels. The relationship between the cushioning coefficient of the aerogels and the maximum static stress is shown in Figure 3e. The smaller the cushioning coefficient, the better the cushioning performance of the aerogel [16]. As can be seen, the cushioning properties of CNGA_50-7_ (4.887) and CNGA_75-7_ (5.039) are better than those of CNGA_25-7_ (5.170), indicating that the addition of glycerin improved the cushioning performances of the aerogels. The maximum static pressure corresponding to the minimum cushioning coefficient of CNGA_75-7_ (0.518 MPa) was higher than that of CNGA_50-7_ (0.186 MPa). Therefore, the application scenarios that match the CNGA_75-7_ have a wide range of stress options, which is more suitable for the actual storage and transportation of fruits and vegetables. The resilience rate of the aerogels is shown in Figure 3f. The CNGA_25-7_, CNGA_50-7_, and CNGA_75-7_ samples all had good resilience, and the average resilience of CNGA_75-7_ was the highest (92.7%). After comprehensive consideration of the cushioning performance and resilience of the aerogel, 75Ink was selected as the 3D-printing ink for further experiments.

### 2.3. Effect of Cross-Linking Time on 3D-Printed Cushioning Aerogels

**Figure 4:**
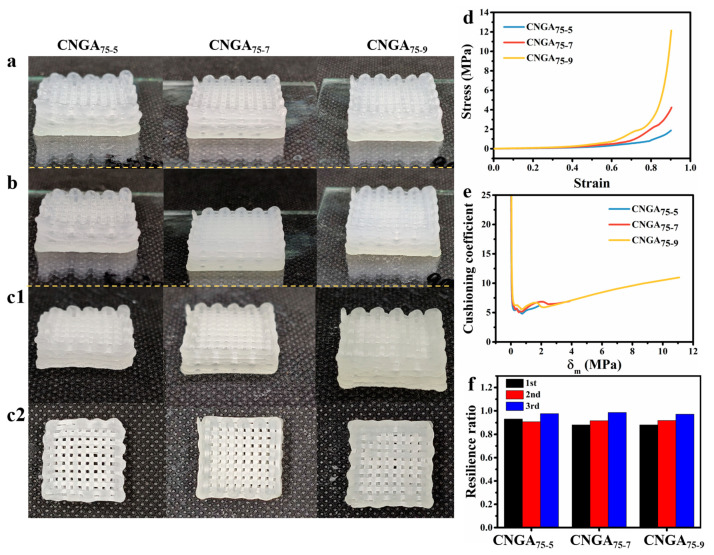
Morphology of 3D-printed samples by 75Ink with different cross-linking time (**a**) before and (**b**) after UV curing and (**c1**,**c2**) after freeze drying. (**d**) Stress–strain curve, (**e**) cushioning curves, and (**f**) thrice compression resilience ratio of samples with different cross-linking time.

Samples with different cross-linking time (before and after UV curing and after lyophilization) are shown in Figure 4a–c. With the extension of cross-linking time, the degree of cross-linking increased, and the color of the samples showed a tendency to turn yellow after cross-linking and freeze-drying. When the cross-linking time reached 9 min, the printed fiber with network structure shrank, and its diameter became thinner.

With the increase of cross-linking degree, the elastic modulus of the aerogel increased, and the stress under the same strain also increased with the increase of cross-linking time (Figure 4d). Although the difference of stress between the samples was small in the low strain range, the slope of the stress–strain curve of the aerogel increased sharply when the strain exceeded 60%. In addition, with the extension of cross-linking time, the minimum cushioning coefficient of the aerogel presented an increasing trend (Figure 4e). Although the cushioning coefficient of CNGA_75-7_ is close to that of CNGA_75-5_, both are lower than that of CNGA_75-9_. In terms of resilience, the samples with different cross-linking time all had excellent resilience, and the average resilience rates were higher than 90% (Figure 4f). In short, with the increase of cross-linking time, the cushioning performance and resilience of the aerogel both showed a decreasing trend. In addition to the excellent resilience and cushioning performance, the cushioning packaging aerogel should also have the strength of shock resistance. Therefore, the CNGA_75-7_-printed sample is more suitable to be the fruit cushioning packaging aerogel.

### 2.4. Characterization of 3D-Printed Cushioning–Antibacterial Packaging Aerogels

**Figure 5:**
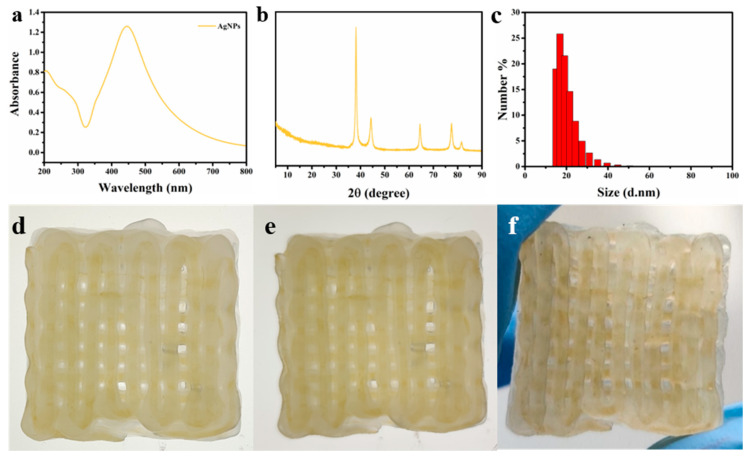
(**a**) Ultraviolet spectrum, (**b**) XRD spectra and (**c**) particle size of silver nanoparticles (AgNPs); photographs of CMC-based 3D-printed aerogel (**d**) before curing, (**e**) after curing, and (**f**) after freeze drying.

After successfully obtaining 3D-printed CMC-based cushioning aerogels, chitosan/AgNPs were immobilized inside the aerogels by coaxial 3D-printing technology to achieve dual functions of cushioning and antibacterial for meeting the practical application requirements. It can be seen that a wide absorption peak in the 400–500 nm region was observed in the ultraviolet spectrum (Figure 5a), indicating that AgNPs have been successfully synthesized [17]. There were five characteristic peaks in the X-ray diffraction spectrum of AgNPs, located at 38.2°, 44.4°, 64.6°, 77.4°, and 81.6°, corresponding to the (111), (200), (220), (311), and (222) crystal planes of AgNPs, respectively (Figure 5c). This is consistent with the standard diffraction peak of silver nanoparticles (JCPDS no.89-3722). Furthermore, the prepared AgNPs had a uniform particle size, with an average of about 20 nm (Figure 5b). As shown in Figure 5d–f, CMC-based 3D-printed cushioning–antibacterial packaging aerogel (CNGA/C–AgNPs) was successfully prepared through coaxial 3D-printing technology. There was a yellow linear region formed by immobilized chitosan/AgNPs in the translucent matrix, and chitosan/AgNPs were embedded in the center of the printed fiber to form a core–shell fiber with a translucent matrix shell and a yellow chitosan/AgNPs core.

As shown in Figure S2, in the FT-IR spectrum of CMC, the wide peak at 3340 cm^−1^ was related to O-H stretching vibration [18]. The FT-IR characteristic peaks of CMC were the symmetric and asymmetric vibrations of carboxylate at 1601 cm^−1^ and 1420 cm^−1^ as well as the C-O-C stretching vibration at 1035–1060 cm^−1^ [19,20]. The characteristic peaks of Cts were at 1640 cm^−1^ (amide I, C=O stretching vibration) and 1547 cm^−1^ (amide II, -NH_2_ bending vibration) [21,22]. The FT-IR characteristic peaks of Cts at 1068 cm^−1^ and 1030 cm^−1^ were the stretching vibration peaks of secondary and primary alcohol hydroxyl groups, respectively [23]. The wide band in chitosan (Cts) spectra at 3000–3650 cm^−1^ was due to the overlap of O-H and N-H stretching vibration. In the FT-IR spectra of CNGA_75-7_ and CNGA/C–AgNPs, the characteristic peak at 1650 cm^−1^ was derived from the C=O stretching vibration and the -CH_2_ symmetric stretching in N, N′-methylenebis(acrylamide) and N-(2-hydroxyethyl) acrylamide [24]. The peak at 2925 cm^−1^ was related to the symmetric stretching of -CH_2_ in N, N′-methylenebis(acrylamide) [24]. The strong peak at 1033 cm^−1^ was due to the plane bending vibration of -OH, which may be related to the addition of glycerol [24]. From the above analysis, it can be seen that each component in the aerogels were recombined successfully.

**Figure 6:**
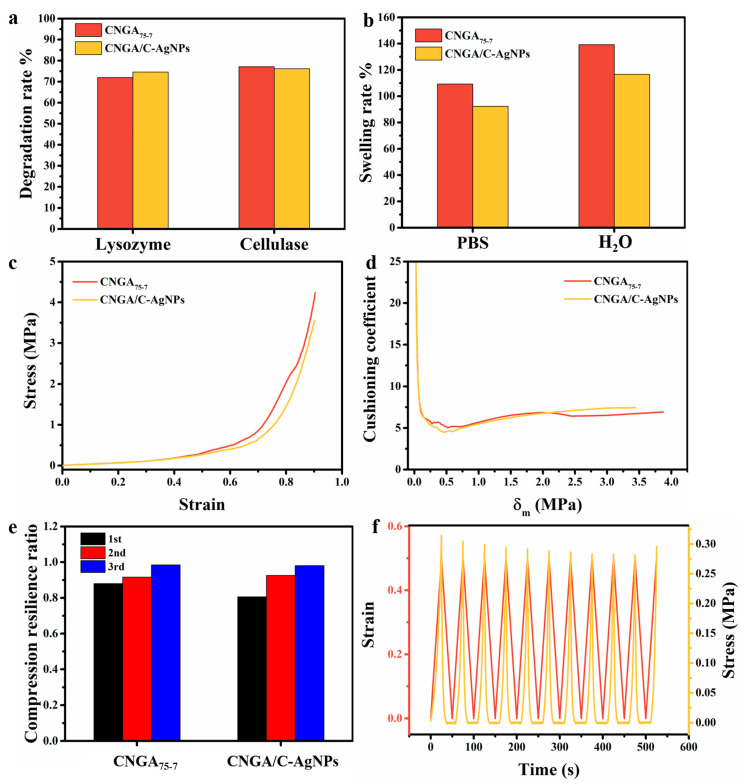
(**a**) Degradation rate and (**b**) swelling rate of aerogels before and after immobilizing AgNPs. (**c**) Stress–strain curve, (**d**) cushioning curves, and (**e**) thrice compression resilience ratio of samples with/without AgNPs; (**f**) compression cycle curve of CNGA/C–AgNPs.

The degradation and swelling properties of packaging aerogels were also investigated (Figure 6). As can be seen from Figure 6a, the degradation rate of CMC-based 3D-printed cushioning–antibacterial packaging aerogels (CNGA/C–AgNPs) in the environment of lysozyme and cellulase reached more than 70% within 14 days, and the embedding of AgNPs had almost no effect on the degradation rate. In addition, as shown in Figure 6b, the swelling rate of CNGA/C–AgNPs and CNGA_75-7_ in H_2_O were 116.67% and 139.15%, respectively. Meanwhile, those in PBS were 92.31% and 109.31%, respectively. The swelling rate of the aerogel (CNGA/C–AgNPs) after embedding AgNPs was lower than that before embedding (CNGA_75-7_). This is because the electrostatic interaction between chitosan and carboxymethyl nanocellulose (CMC), and the filling effect of AgNPs in the inner layer of aerogel make it difficult for water to invade. This property is conducive to the moisture stability of the packaging aerogel.

### 2.5. The Cushioning Performance of 3D-Printed Cushioning–Antibacterial Packaging Aerogels

The cushioning performance of CMC-based 3D-printed dual-functional packaging aerogels was evaluated by a universal material testing machine. As shown in Figure 6c,d, the minimum cushioning coefficient of the samples decreased from 5.04 to 4.29 after AgNPs were immobilized in the fibers of packaging aerogel. Compared with CNGA_75-7_, the cushioning performance of CNGA/C–AgNPs was significantly improved. The toughening effect of chitosan/AgNPs and the electrostatic interaction between chitosan and carboxymethyl nanocellulose (CMC) is conducive to the absorption and dissipation of energy. In addition, the cushioning coefficient–maximum static pressure curve of CNGA/C–AgNPs sample was similar with that of commercial polyethylene foam (EPE), indicating that they had similar cushioning properties. The average compression resilience ratio of the CNGA/C–AgNPs sample was more than 90% (Figure 6e), and it had good compression stability (Figure 6f).

### 2.6. Ag Release Behavior and Bacterial Inhibition Performance of 3D-Printed Cushioning–Antibacterial Packaging Aerogels

**Figure 7:**
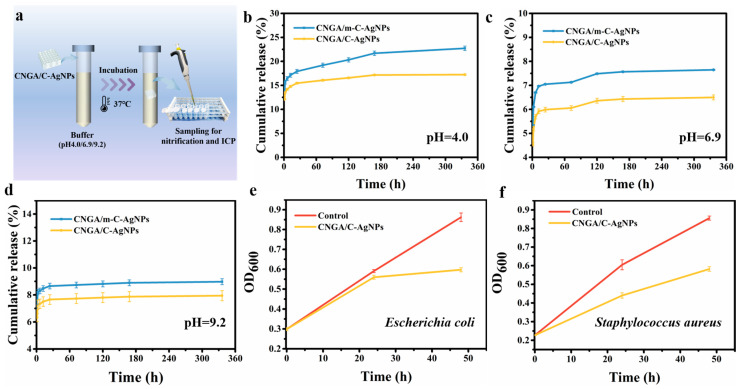
(**a**) Schematic diagram of Ag release experiment. Cumulative release rates of silver nanoparticles under different pH: (**b**) pH 4.0, (**c**) pH 6.9, and (**d**) pH 9.2. OD_600_ curves of (**e**) *E. coli* and (**f**) *S. aureus*.

In order to investigate the difference of immobilization effect between the shell core structure formed by coaxial 3D printing and the direct blending printing, the Ag release behavior of the samples obtained by these two printing methods was analyzed (Figure 7a). The shell material (75Ink) and core solution (Chitosan/AgNPs) of CNGA/C–AgNPs were blended to form a new composite, and then, it was used as the printing ink to obtain a CNGA/m-C–AgNPs sample through uniaxial 3D printing. According to Figure 7, in different pH environments (pH = 4.0, 6.9, 9.2), the cumulative release of AgNPs of the 3D-printed cushioning–antibacterial packaging aerogel (CNGA/C–AgNPs) was lower, indicating that the coaxial 3D printing had a good immobilization efficiency for AgNPs, and the packaging aerogel had a good security. It is worth noting that the AgNPs’ cumulative release of CNGA/C–AgNPs samples in an acidic environment (Figure 7b) was significantly higher than that in neutral and alkaline environments (Figure 7c,d). This was due to the protonization of chitosan by H^+^ in an acidic environment and the dissolution of the chitosan, which promoted the release of AgNPs. The release rate of the CNGA/C–AgNPs sample tended to be slow after 48 h, while the CNGA/m-C–AgNPs sample showed a long rapid release period of AgNPs, indicating that the immobilization effect of coaxial 3D printing remarkably controlled the release of AgNPs.

Herein, common foodborne pathogenic bacteria [25], *Escherichia coli* and *Staphylococcus aureus*, were selected to evaluate the bacterial inhibition activity of CMC-based 3D-printed cushioning–antibacterial packaging aerogel. In the presence of CNGA/C–AgNPs, the OD_600_ of bacterial suspension was significantly reduced compared with the control group, indicating that CNGA/C–AgNPs effectively inhibited the proliferation and activity of bacteria. Compared with the control group, with the increase of time, the activity of bacterial inhibition of the material against *Escherichia coli* increased, and after 24 h, the activity of bacterial inhibition became higher than that in the first 24 h (Figure 7e). The activity of bacterial inhibition against *Staphylococcus aureus* had the same trend (Figure 7f). It was because the amount of released antibacterial agents increased with time. In addition, after 24 h, the inhibitory effect of the material on *Staphylococcus aureus* was better than that of *Escherichia coli*, which may be related to the structural difference between Gram-negative bacteria and Gram-positive bacteria [26].

## 3. Materials and Methods

### 3.1. Materials

Carboxymethyl nanocellulose (CMC) (carboxyl content: 1.08 mmol/g) was purchased from Mujinglin Biotechnology Co., Ltd. (Tianjin, China); chitosan was obtained from Haidebei Marine Biotechnology Co., Ltd. (Jinan, China); tea polyphenol, N, N′-methylenebis(acrylamide), and Irgacure 2959 were purchased from MackLin Biochemical Technology Co., Ltd. (Shanghai, China); silver nitrate and N-(2-hydroxyethyl) acrylamide were purchased from Aladdin Biochemical Technology Co., Ltd. (Shanghai, China); *Escherichia coli* and *Staphylococcus aureus* were provided by Guangzhou Institute of Microbiology (Guangzhou, China). All other reagents were analytical grade.

### 3.2. Preparation of Silver Nanoparticles

First, 0.1 g of tea polyphenol was dissolved in 10 mL of deionized water to obtain tea polyphenol solution. Then, 0.34 g of silver nitrate was dispersed in 45 mL of deionized water, and then ammonia was added drop by drop until the solution became transparent. Then, 5 mL of the prepared tea polyphenol solution was added to the silver ammonia solution under stirring, and then, it was reacted at 60 °C for 1 h to obtain a silver nanoparticles antimicrobial agent.

### 3.3. Preparation of 3D-Printing Ink

Shell ink, 0.2 g of Irgacure 2959, and 0.32 g of N, N′-methylenebis(acrylamide) were mixed with 8 mL of 75 vol% glycerine solution, respectively, and heated in a water bath at 65 °C until completely dissolved. Then, 10 g of carboxymethyl nanocellulose (CMC) were mixed with 4 mL of Irgacure 2959 solution and 4 mL of N, N′-methylenebis(acrylamide) solution under stirring. Then, 1.2 g of N-(2-hydroxyethyl) acrylamide was added into the mixture with stirring. The obtained 3D-printing ink was named as 75Ink.

When the 75 vol% glycerine solution was replaced by the same amount of 50 vol%, 25 vol% glycerin solution, or DI water, the obtained 3D-printing inks were named as 50Ink, 25Ink, and 0Ink, respectively.

Core solution: 500 μL of prepared silver nanoparticles (AgNPs) were dispersed into 5 mL of 0.5 wt % chitosan solution containing 1% acetic acid to be the core solution of coaxial 3D printing.

### 3.4. Preparation of 3D-Printed Cushioning Packaging Aerogels

The 3D-printed CMC-based cushioning packaging aerogel was prepared by Y&D7300N 3D printer (Yida TEC CO., Dongguan, China) through uniaxial printing technology. Inks with different glycerol content (25Ink, 50Ink, 75Ink) were extruded through 16 G needle (1.26 mm) to obtain the corresponding samples. The 3D-printed samples were irradiated by UV curing device for 7 min followed by freeze drying, which were respectively named CNGA_25-7_, CNGA_50-7_, and CNGA_75-7_.

With the UV curing time modulated for 5 min and 9 min, the obtained samples were named CNGA_75-5_ and CNGA_75-9_, respectively.

### 3.5. Preparation of 3D-Printed Cushioning–Antibacterial Packaging Aerogels

CMC-based cushioning–antibacterial packaging aerogels were prepared by coaxial printing using a Y&D7300N 3D printer (Yida TEC CO., Donggaun, China). During the printing process, 75Ink was extruded through a coaxial needle (17 G/22 G) at a speed of 4 mm/s. The core solution (Cts/AgNPs) was extruded through an LSP04-1A syringe pump (Longer Pump, Baoding, China) (3 mL/h). After printing, UV curing was carried out for 7 min and then freeze dried to obtain CMC-based cushioning–antibacterial packaging aerogel (CNGA/C–AgNPs).

Then, 75Ink was mixed with the core solution (Cts/AgNPs) to obtain the sample CNGA/m-C–AgNPs through uniaxial 3D printing, which was followed by UV curing and freeze drying. CNGA/m-C–AgNPs acted as the control group for the Ag release experiment to investigate the influence of the embedding effect of coaxial 3D printing on the release behavior of AgNPs.

### 3.6. Rheological Test of 3D-Printing Inks

The rheological test was carried out by a ARES-G2 rheometer (TA Instruments, New Castle, DE, USA) equipped with 40 mm parallel plates.

Flow Sweep: The flow sweep measurements were conducted at the shear rate range of 10^−2^–10^2^ s^−1^ at 25 °C, 5 points per decade. The samples reach the equilibrium temperature for 180 s prior to the measurement.

Oscillation Amplitude: The test was conducted at stain from 0.01% to 100% with angular frequency of 6.28 rad/s at 25 °C.

### 3.7. Characterization of 3D-Printed Packaging Aerogels

UV-vis spectra were measured by UV-1800 (Shimazu, Kyoto, Japan). The particle size distribution was measured using the SZ-100Z Nanoparticle Analyzer (Horiba, Kyoto, Japan). Scanning electron microscope (SEM) micrographs were obtained by a Merlin (Zeiss, Germany) microscope. FT-IR spectrometer (VERTEX 70, Bruker, Karlsruhe, Germany) recorded from 4000 to 400 cm^−1^ and X-ray diffraction spectrometer (X’pert Powder, PANalytical, Almelo, Netherlands) worked with Cu Kα radiation in the scan range of 2θ from 5° to 90°.

### 3.8. Cushioning and Resilience Performance of 3D-Printed Packaging Aerogels

The cushioning and resilience performance of the samples were measured by an INSTRON 5565 electronic universal material tester (Instron, Boston, MA, USA).

Cushioning performance: The data collected in the static compression experiment (compression rate: 5 mm/min, final strain: 90%) were calculated according to the following formulas to evaluate the cushioning performance of the aerogels. The smaller the cushioning coefficient C, the more energy is absorbed per unit volume of the materials and the higher the cushioning efficiency. The minimum value of the cushioning factor usually corresponds to the best application scenario of the material.

(1) $$e=\overset{\varepsilon }{\underset{0}{\int }}\delta d\varepsilon$$

(2) C=δe

Among them, *δ* is the compressive stress of the sample, and *ε* is the compressive strain of the sample.

Resilience performance: The test was carried out as described by Li et al. [27] with slight modification. The loading speed during the compression process was 12 mm/min. Afterwards, the samples were compressed to 50% strain and held for 3 min. Then, the thickness of the samples was measured after 10 s of recovery. The compression test was repeated three times for each sample. The resilience rate of the sample was calculated according to the following formula.

(3) Rj=tj−(Ti2)Ti2

where R_j_ is the resilience rate of the jth time, j = 1, 2, 3; T_j_ and T_j_ are respectively the thickness before and after the jth compression of the samples, j = 1, 2, 3.

### 3.9. Biodegradation and Swelling Performance of the 3D-Printed Packaging Aerogels

Biodegradation performance: A certain mass (0.2 g) of sample was immersed in 10 mL PBS containing 2 × 10^4^ U/mL lysozyme or 150 U/mL cellulase, respectively, incubated at 37 °C for 14 days, and then taken out, freeze dried, and weighed. The degradation rate of sample (*D*) was calculated by the following formula:

(4) $$D\%=\frac{W_{0}-W_{t}}{W_{0}}\;\times \;100\%$$

where *W*_0_ (g) is the initial sample weight, and *W_t_* (g) is the sample weight after 14 days.

Swelling performance: The swelling performance of the aerogels was measured by the following method: The aerogels were immersed in PBS and deionized water respectively, and taken out after keeping at 25 °C for 24 h. The liquid on the surface was dried with a filter paper and weighed. The swelling rate (S) can be calculated according to the following formula:

(5) $$S\%=\frac{m_{1}-m_{0}}{m_{0}}\;\times \;100\%$$

where m_0_ (g) is initial sample weight, and m_1_ (g) is the sample weight after swelling for 24 h.

### 3.10. The AgNPs Release Behavior of 3D-Printed Packaging Aerogels

The 3D-printed CMC-based cushioning–antibacterial packaging aerogel was immersed in 10 mL of buffer solution (pH = 4.0, 6.9, 9.2) and incubated at 37 °C, which was followed by taking out 0.5 mL of sample solution and adding the same amount of fresh buffer at specific times (*t* = 1, 2, 6, 12, 24, 72, 120, 168, 336 h). Then, 4.5 mL of nitric acid solution (1M) was added to the sample solution for nitrification, and the silver content was detected by PE 8300 inductively coupled plasma emission spectrometer (PerkinElmer, Waltham, MA, USA).

### 3.11. Bacterial Inhibition Activity of 3D-Printed Packaging Aerogels

The bacterial inhibition activity of 3D-printed CMC-based cushioning–antibacterial packaging aerogel was evaluated by the following methods: Firstly, the colonies of *Escherichia coli* and *Staphylococcus aureus* were scraped and dispersed in the LB liquid medium to prepare the bacterial suspension of *Escherichia coli* and *Staphylococcus aureus*, respectively. Certain mass samples (0.2 mg) were soaked in the bacterial suspension and cultured at 37 °C. Then, the bacterial suspension was taken out at specific times (0, 12, 24 h) for measuring the OD_600_ value by Multiskan Go enzyme spectrometer (Thermo Scientific, Waltham, MA, USA). The bacterial inhibition activity of the samples was analyzed by the difference of OD_600_ value between the experimental group and the control group.

## 4. Conclusions

In this study, CMC-based cushioning–antimicrobial dual-function food packaging aerogel (CNGA/C–AgNPs) was successfully prepared by using carboxymethyl nanocellulose as the matrix, glycerol/acrylamide derivative as supplemented, and AgNPs were immobilized as the core layer through coaxial 3D-printing technology. The prepared CNGA/C–AgNPs packaging aerogel has good cushioning and resilience performance. The hollow structure constructed by coaxial 3D-printing technology has a good immobilization efficiency for AgNPs. Additionally, the packaging aerogel has an obvious bacterial inhibition effect on *E. coli* and *S. aureus*. Meanwhile, the packaging aerogel with cushioning–antimicrobial dual function is biodegradable. Hence, coaxial 3D-printing technology has great application potential in the construction of food packaging aerogels, especially cushioning packaging for the storage and transportation of fruits and vegetables.

## Data Availability

Not applicable.

## Supplementary Materials

The following are available online, Figure S1: Microscopic morphology of (a1, a2) CNGA_25-7_; (b1, b2) CNGA_50-7_; (c1, c2) CNGA_75-7_after freeze-drying, Figure S2: FT-IR spectra of CMC, Cts, CNGA_75-7_ and CNGA/C-AgNPs.

## Sample Availability

Samples of the compounds are not available from the authors.
