# Supplementary material for: 3D-Printed Nanocellulose-Based Cushioning–Antibacterial Dual-Function Food Packaging Aerogel
Source: Molecules. 2021 Jun 10;26(12):3543. doi: 10.3390/molecules26123543 (PMC8228327; doi:10.3390/molecules26123543)
Supplement: Supplementary file 1 [file molecules-26-03543-s001.zip › molecules-1244806-supplementary.pdf]

Supplementary Materials

3D printed nanocellulose-based cushioning-antibacterial dual-function food packaging areogel

Wei Zhou[1], Jiawei Fang[1], Shuwei Tang, Zhengguo Wu* and Xiaoying Wang

State Key Laboratory of Pulp and Paper Engineering, South China University of Technology, 381 Wushan Road, Tianhe District, Guangzhou, 510640, China

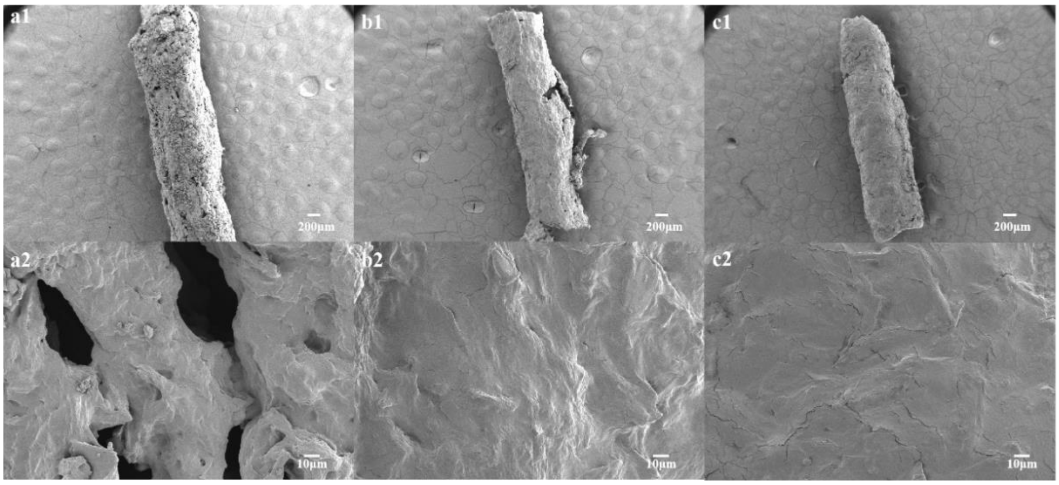

Figure S1. Microscopic morphology of (**a1**, **a2**) $CNGA_{25\text{-}7}$; (**b1**, **b2**) $CNGA_{50\text{-}7}$; (**c1**, **c2**) $CNGA_{75\text{-}7}$after freeze-drying.

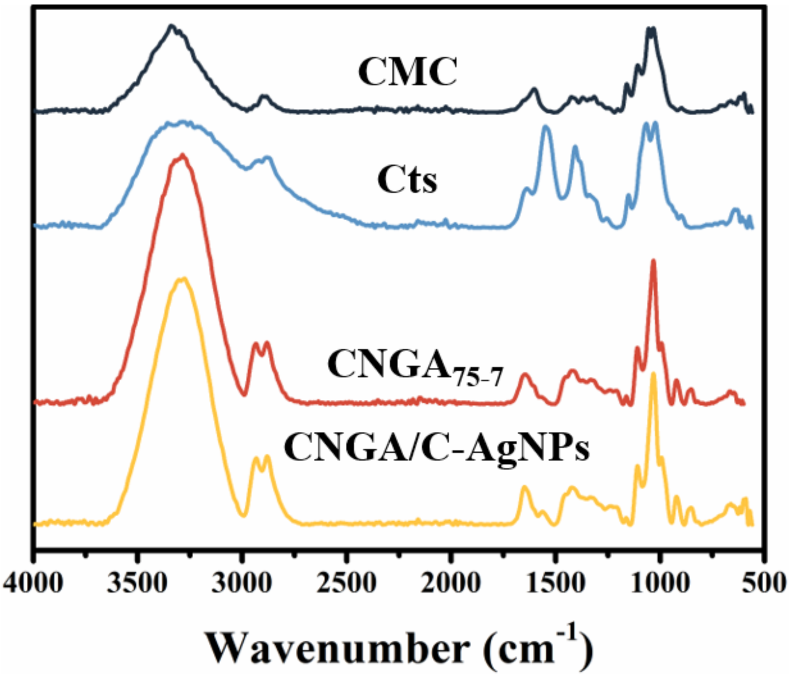

Figure S2. FT-IR spectra of CMC, Cts, $CNGA_{75\text{-}7}$ and CNGA/C-AgNPs.
